# HDAC inhibitors with potential to overcome drug resistance in castration-resistant prostate cancer

**DOI:** 10.20517/cdr.2021.105

**Published:** 2022-01-04

**Authors:** Bernhard Biersack, Bianca Nitzsche, Michael Höpfner

**Affiliations:** ^1^Organic Chemistry Laboratory, University of Bayreuth, Bayreuth 95440, Germany.; ^2^Institute of Physiology, Charité-Universitätsmedizin Berlin, Berlin 10117, Germany.

**Keywords:** Histone deacetylases, HDAC inhibitors, castration-resistant prostate cancer, drug resistance

## Abstract

Epigenetic mechanisms play an important role in the development and persistence of cancer, and histone deacetylase (HDAC) inhibitors are promising anticancer drugs targeting epigenetic modes. Efficient anticancer drugs for the treatment of castration-resistant prostate cancer (CRPC) are sought, and approved HDAC inhibitors have shown promising results on the one hand and severe drawbacks on the other hand. Hence, ways to break the drug resistance mechanisms of existing HDAC inhibitors as well as the design of new promising HDAC inhibitors which can overcome the disadvantages of the classic HDAC inhibitors are of great importance. In this work, HDAC inhibitors with the potential to become a mainstay for the treatment of CRPC in the future as well as suitable combination treatments of HDAC inhibitors with other anticancer drugs leading to considerable synergistic effects in treated CRPCs are discussed.

## INTRODUCTION

More than 1.2 million new cases of prostate cancer cases are reported per year worldwide (2018) rendering prostate cancer the second most diagnosed cancer in men along with a rising incidence over the last years^[1]^. The formation of metastases dramatically reduces the survival rates in prostate cancer patients^[2]^. Advanced forms of prostate cancer are commonly treated by hormone therapy, either by surgical castration or by chemotherapy targeting hormone release^[3]^. However, patients develop castration-resistant prostate cancer (CRPC) forms within a median time of two years after treatment, which pose a life-threatening danger to affected patients with distinctly poorer prognosis^[4]^. CRPC can occur in metastatic (mCRPC) and non-metastatic (M0CRPC) forms. Clinical trials using anti-androgen therapies with drugs such as apalutamide, enzalutamide, or darolutamide showed promising outcomes and significantly prolonged metastasis-free survival in M0CRPC patients^[5-7]^. Treatment options for mCRPC patients include taxanes such as docetaxel and cabazitaxel as well as various anti-androgens (bicalutamide and enzalutamide)^[8]^. The development of the selective CYP17 inhibitor abiraterone and its prodrug abiraterone acetate, specifically blocking androgen synthesis and androgen receptors via CYP17 enzyme inhibition, has led to another valuable first-line treatment option for mCRPC patients^[9]^.

Chromatin remodeling is a crucial mechanism in proliferating cells and affects transcription, replication, and DNA repair processes. The nucleosome is the basic unit of chromatin and consists of the positively charged histone core octamer proteins (H2A, H2B, H3, and H4, each twice per octamer) wrapped by DNA of 147 base pairs and one attached H1 histone^[10,11]^. *N*-terminal lysines of the histones are crucial for their DNA interaction, and the charge of these lysines is regulated by cellular *N*-acetylation processes. Acetylation of histones reduces their interaction with DNA leading to chromatin decondensation and gene transcription, while histone deacetylation silences gene expression in the densely packed chromatin^[12]^. Histone modifying enzymes such as histone acetyl transferases (HATs), which catalyze lysine acetylation, and histone deacetylases (HDACs), which catalyze the removal of acetyl groups from target protein lysines, play an eminent role in these processes and, in particular, inhibitors of HDACs were developed as anticancer agents^[13]^. The scope of HDAC inhibitors is promising since they also affect the acetylation state of non-histone proteins, such as p53, Hsp90, STAT3, and NF-κB, and regulate the stability and/or DNA binding properties of these non-histones in this way with significant effects on, for instance, gene transcription, cell division, signal transduction, DNA repair, and apoptosis induction^[14,15]^. In terms of HDACs and prostate cancers, the HDAC-based regulation of the androgen receptor (AR) via the acetylation state of Hsp90 is especially interesting and of high relevance for the design of HDAC inhibitor-based prostate cancer therapies for patients with poor prognosis (see below)^[15,16]^. *N*-Methylation of lysines is another regulatory modification of histones controlled by lysine demethylases (KDMs), which can have silencing and activating effects on gene transcription in cancers^[17]^. KDMA1 (LSD1) has oncogenic functions in prostate cancers by AR co-activation, suppression of p53, and activation of c-Myc expression, and the development of KDM inhibitors as anticancer agents (e.g., cyclopropylamines) paves the way to promising epigenetic treatment options. The combination of KDM1A and HDAC inhibitors showed synergistic effects on glioblastoma cells^[17]^. The HDAC inhibitor vorinostat also inhibited EZ2H and H3K4 demethylases in the micromolar concentration range^[17-19]^.

A few HDAC inhibitors have already reached clinical application and are used for the treatment of multiple myeloma (panobinostat) and T-cell lymphoma (vorinostat, romidepsin, and belinostat); however, the performance of these first-generation HDAC inhibitors in solid tumors is rather poor^[20]^. Nevertheless, HDACs can be suitable anti-prostate cancer drug targets. HDAC1, for instance, was found to be upregulated in hormone refractory prostate cancer^[21]^. HDAC1, -2, and -3 expression was associated with Ki-67-positive prostate cancer cell fractions, and high HDAC2 levels were detected in patients with reduced disease-free survival^[22]^. HDACs are necessary for functional AR signaling in prostate cancers, and HDAC inhibitors such as vorinostat and panobinostat blocked AR function by reducing AR expression and inhibiting coactivator/RNA polymerase II complex formation after AR binding to its DNA target element^[23]^. Resistance mechanisms of prostate cancers to HDAC inhibitor treatment comprise the upregulation of detoxifying P-glycoprotein (P-gp) transporters, increased expression of HDAC enzymes, suppression of HAT enzymes, and upregulation of tumorigenic p21 (p21 is usually a tumor suppressor, but oncogenic functions of p21 were reported upon HDAC treatment)^[24]^. HDAC inhibitors also induced epithelial-to-mesenchymal transition (EMT) in prostate cancers, which might be another reason for the drawbacks of HDAC inhibitors in solid tumors^[25]^.

HDAC enzymes can be subdivided into four classes. Classes I (HDAC1, -2, -3, -8), IIa (HDAC4, -5, -7, and -9), IIb (HDAC6 and -10), and IV (HDAC11) are metal-dependent with a catalytic zinc ion in the active site, while Class III HDACs (sirtuins) are not dependent on metals^[26,27]^. Due to the catalytic zinc ion of Class I, II, and IV HDACs, many HDAC inhibitors have a molecular zinc-binding group (ZBG) such as carboxylates, hydroxamic acids, benzamides, substituted ketones, mercaptoacetamides, or depsipeptides^[28]^. The modular composition of most HDAC inhibitors comprising a ZBG and a capping group connected by a linker allows the fine-tuning of HDAC inhibitors in terms of pharmacokinetics and anticancer activity^[28]^. Several dual or multimodal HDAC inhibitors have been reported, which showed improved anticancer activities especially against solid tumors^[29,30]^. This development is still ongoing and offers interesting compounds for the treatment of drug-resistant CRPC where first-generation HDAC inhibitors failed to perform.

This review provides the current state of the art of HDAC inhibitors under investigation in CRPCs and the potential of HDAC inhibitors to overcome drug resistance in this severe cancer form. A focus is set on next generation HDAC inhibitors with improved biological properties.

## HDAC INHIBITORS IN CRPC CLINICAL TRIALS

**Figure 1:**
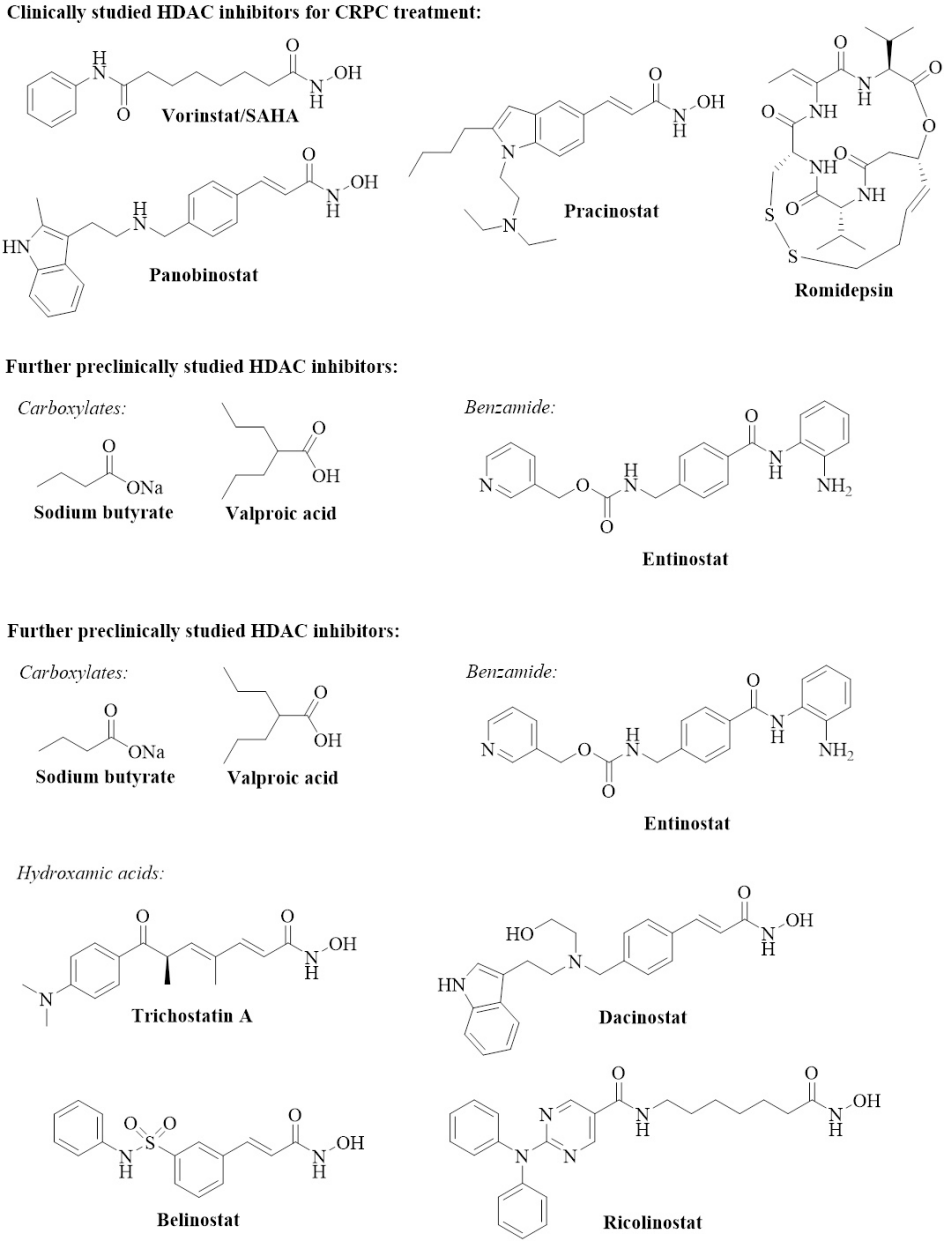
Structures of clinically and preclinically tested HDAC inhibitors. HDAC: Histone deacetylase; CRPC: castration-resistant prostate cancer.

Several clinical trials with HDAC inhibitors were carried out to monitor advantages and problems of the usage of HDAC inhibitors in the clinics. The clinical trials of HDAC inhibitors for the treatment of castration-resistant prostate cancer diseases were recently reviewed, and only the main outcomes are summarized in Table 1 and Figure 1^[24,31]^.

**Table 1:** CRPC clinical trials of HDAC inhibitors

**Drug(s)**	**Trial**	***n***	**Dosage**	**Outcome**
Romidepsin^[32]^	Phase 2	35	30 mg/m^2^ (i.v.)	PR = 2; SD = 11; PD = 22
Vorinostat^[33]^	Phase 2	27	400 mg/day (p.o.)	SD = 2; PD = 13
Panobinostat^[34]^	Phase 2	35	20 mg/m^2^ (i.v.)	SD = 4; PSA reduction = 5; PD = 29
Panobinostat (+ Docetaxel and Prednisone)^[35]^	Phase 1	8	15 or 20 mg 3× per week (p.o.)	PSA reduction = 5
Panobinostat (+ Bicalutamide)^[36]^	Phase 1	9	60, 90, or 120 mg/week (p.o.)	Stable PSA = 3; PSA reduction = 4
Panobinostat (+ Bicalutamide)^[37]^	Phase 2	29	40 mg triweekly for 2 weeks (p.o.)	rPF = 47.5%; median time to rP = 33.9 weeks
Pracinostat^[38]^	Phase 2	32	60 mg, 3× per week (p.o.)	SD = 7; PSA reduction = 2

PR: Partial response; SD: stable disease; PD: progressing disease; PSA: prostate-specific antigen; rP: radiographic progression; rPF: radiographic progression-free.

The depsipeptide romidepsin underwent a phase 2 clinical trial with 35 CRPC patients. Intravenous romidepsin treatment (13 mg/m^2^) led to radiological partial response in two patients and stable disease in 11 patients, while 22 patients showed progressive disease. Although no grade 4 toxicities were observed, 11 patients had to abandon the trial early, and, thus, romidepsin was not recommended for further phase 3 trials for CRPC^[32]^.

The hydroxamic acid vorinostat (400 mg/day, p.o.) showed drug-induced toxicities in 11 out of 27 CRPC patients during a phase 2 trial, who had to be removed from the study, while its anticancer activity was poor: only 2 patients had stable disease and 13 patients showed disease progression^[33]^. It seems that high IL-6 levels contributed to the failure of vorinostat in the patients with progressive CRPC disease.

Similar to romidepsin, the hydroxamate derivative panobinostat underwent a phase 2 trial with 35 CRPC patients. Intravenous panobinostat administration (20 mg/m^2^) led to PSA (prostate-specific antigen) reduction in only 14% of the patients and disease progression in 29 out of 35 patients^[34]^. A phase 1 study of orally administered panobinostat compared with oral panobinostat in combination with docetaxel and prednisone showed only PSA reduction effects in five out of eight CRPC patients of the combination arm of the trial^[35]^. The oral panobinostat monotherapy showed no effects, and, thus, future studies should include combination therapies with suitable anticancer drugs. A combination study with oral panobinostat and bicalutamide (phase 1/2 trial) in nine CRPC patients led to stable PSA levels in three patients and to PSA reduction of more than 50% in two patients^[36]^. The phase 2 trial of oral panobinostat (40 mg triweekly for two weeks) with oral bicalutamide showed promising results in CRPC patients, who were resistant to second-line antiandrogen therapy (2ndLAARx)^[37]^. This combination treatment was well tolerated and led to a distinct number of radiographic progression-free patients (47.5%) and prolonged median time to radiographic progression (rP) in treated CRPC patients (33.9 weeks). In addition, five patients showed PSA decline of more than 30%. The results indicate that panobinostat plus bicalutamide overcomes androgen resistance in a considerable number of CRPC patients. It is remarkable that panobinostat was less toxic to patients than other HDAC inhibitors such as romidepsin and vorinostat.

Pracinostat, which has received orphan drug status by the FDA for the treatment of AML, was also studied in a clinical phase 2 trial with 32 CRPC patients. Although only two patients displayed PSA reductions of more than 50%, the drug (60 mg, 3× per week, p.o.) was well tolerated, led to stable disease in 22% of the patients, and reduced the number of circulating tumor cells in nine patients^[38]^.

In brief, the cinnamoyl derivatives panobinostat and pracinostat showed improved toxicity profiles and led to certain antitumor responses in CRPC patients, in particular, when combined with other drugs such as docetaxel or bicalutamide (in the case of panobinostat). In contrast, vorinostat and romidepsin showed drug-induced toxicities in a considerable number of patients who had to stop treatment with these drugs because of them. The relatively meager responses caused by the mentioned first-generation HDAC inhibitors in CRPC patients when applied as a monotherapy is the reason why no phase 3 studies in CRPC patients have been completed for these drugs until today.

## STRATEGIES TO IMPROVE HDAC INHIBITOR ACTIVITIES IN CRPC

To overcome the clinical drawbacks of the first-generation HDAC inhibitors, a thorough elucidation of HDAC inhibitor mechanisms apart from or in consequence of HDAC inhibition is necessary, in particular, in combination with other anticancer drugs. In addition, the development of new tuned HDAC inhibitors appears to be promising in terms of improved clinical outcome. The most relevant research outcomes are provided below.

### HDAC inhibitor mechanisms beyond HDAC

**Figure 2:**
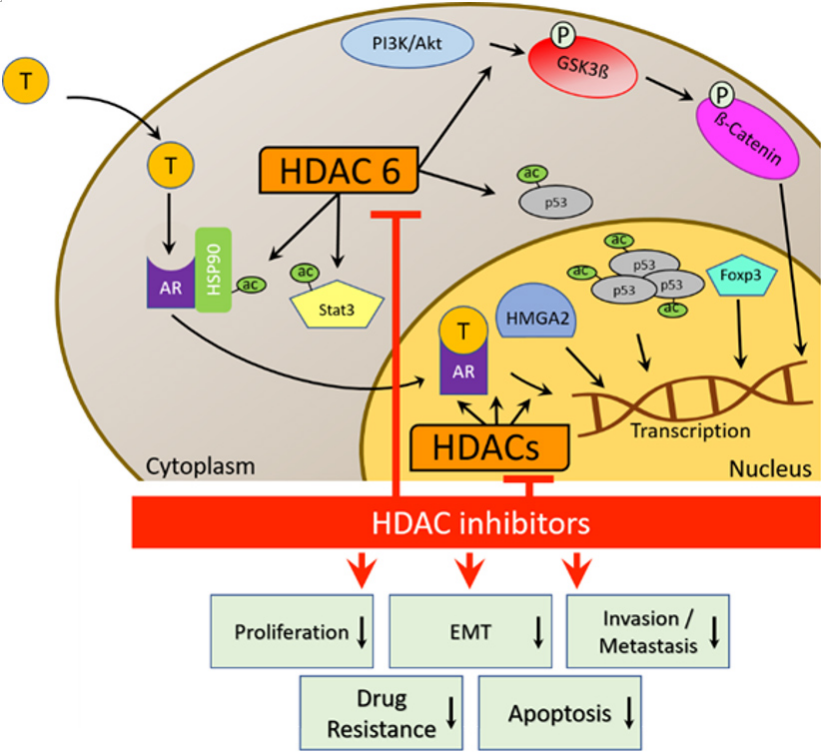
HDAC network and cellular effects of HDAC inhibitors in CRPC. HDAC: Histone deacetylase; HDACi: HDAC inhibitor; T: testosterone; Hsp90: heat shock protein 90; AR: androgen receptor; STAT3: signal transducer and activator of transcription 3; HMGA2: high mobility group AT-hook 2; EMT: epithelial-to-mesenchymal transition.

HDAC inhibition can lead to certain downstream effects in prostate cancer cells [Figure 2, Table 2]. The chemical structures of the investigated compounds are shown in Figure 1. The endogenous HDAC1 inhibitor protein maspin (mammary serine protease inhibitor), which was induced by the HDAC inhibitor entinostat, suppressed AR expression in prostate cancers and led to sensitization of androgen-sensitive LNCaP and castration-resistant 22Rv1 prostate cancer cells to enzalutamide treatment^[39]^.

**Table 2:** HDAC inhibitor effects beyond HDAC inhibition in prostate cancers

**HDAC inhibitor**	**Effect(s)**	**Cell lines/tumor models**
Belinostat^[41]^	Hsp90, AR, and GSK-3β suppression	LNCaP, C4-2B, 22Rv1
Dacinostat^[42]^	AR degradation by high acetyl-Hsp90, Akt inactivation	LNCaP
Entinostat^[39]^	AR suppression, enzalutamide sensitization	LNCaP, 22Rv1
Entinostat^[43]^	IFNγ production, suppressed Foxp3, upregulated acetyl-STAT3, increased survivin vaccine activity	CR Myc-CaP
Panobinostat^[46]^	HMGA2 suppression, EMT formation, increase of acetyl-p53 and acetyl-AR	PKV, MES-like cells/tumors
Trichostatin A^[44]^	Upregulated FGF8 and NF-κB	PC3M
Trichostatin A and sodium butyrate^[47]^	Upregulated CD133	Prostate cancer derived primary endothelial cultures

Hsp90: Heat shock protein 90; AR: androgen receptor; IFNγ: interferon-γ; STAT3: signal transducer and activator of transcription 3; CR: castration-resistant; HMGA2: high mobility group AT-hook 2; EMT: epithelial-to-mesenchymal transition; MES: mesenchymal; PC3M: metastatic PC3 cells; PKV: *Pten*^L/L^, *Kras*^G12D/+^, *Vim-GFP*.

Acetylation of Hsp90 is regulated by cytoplasmic HDACs such as HDAC6, while Hsp90 regulates AR activity and stability in prostate cancers^[40]^. Hormone treatment induced castration-resistant phenotype and upregulated HDAC6 expression in prostate cancer cells. Belinostat (PXD101) suppressed Hsp90 activity, GSK-3β, and AR function by HDAC6 inhibition and prevented the development of castration-resistant phenotype in prostate cancer cells. In addition, belinostat (40 mg/kg bid, i.p.) showed improved activities in 22Rv1 CRPC xenograft models in combination with the androgen antagonist bicalutamide (50 mg/kg, p.o.)^[41]^. Dacinostat (LAQ824), a close analog of panobinostat, suppressed the Hsp90 client protein AR in LNCaP prostate cancer cells by an increase of acetylated Hsp90 levels accompanied by Hsp90-AR dissociation and AR decomposition^[42]^.

Dacinostat also inactivated Akt by disruption of HDAC-PP1 (protein phosphatase 1) complexes, leading to the dephosphorylation of Akt proteins in PC3 prostate cancer cells^[42]^. Consequently, factors associated with Akt signaling were regulated by HDAC inhibitors. The Class I HDAC inhibitor entinostat (5 mg/kg/day, 5 days per week, p.o.) augmented distinctly the anticancer activity of the survivin peptide vaccine SurVaxM as well as IFNγ (interferon-γ) production in Myc-expressing CRPC mouse models (CR Myc-CaP). Entinostat upregulated acetyl-STAT3 accompanied by suppression of Foxp3 in cancer cells, which was suggested as its possible mode of action^[43]^. As part of its hormone-independent prostate cancer promoting effect, the natural HDAC inhibitor trichostatin A (TSA) increased the transcriptional activity of NF-κB in PC3M prostate cancer cells associated with upregulation of fibrose growth factor FGF8^[44]^. Thus, NF-κB suppressing agents might be promising combination partners of entinostat in terms of CRPC eradication. The PI3K/Akt signaling pathway is also involved in EMT induction^[45]^. EMT processes upregulate HMGA2. HMGA2 is a protein crucial for the regulation of gene expression by binding to AT-rich regions of DNA, which is the site where a replication complex is formed and the DNA synthesis is initiated. Interestingly, panobinostat was shown to suppress HMGA2 associated with reduced EMT formation and cell stemness. In addition, panobinostat increased the acetyl-p53 and acetyl-AR levels and prevented mCRPC formation *in vivo* at a dose of 10 mg/kg for five days per week^[46]^. In terms of cancer stem cell markers, the HDAC inhibitors TSA and sodium butyrate upregulated the expression of the surface stem cell marker CD133 in prostate cancer derived primary epithelial cultures, which was based on chromatin relaxation leading to gene expression^[47]^.

### Promising combinations of HDAC inhibitors with anticancer drugs

**Figure 3:**
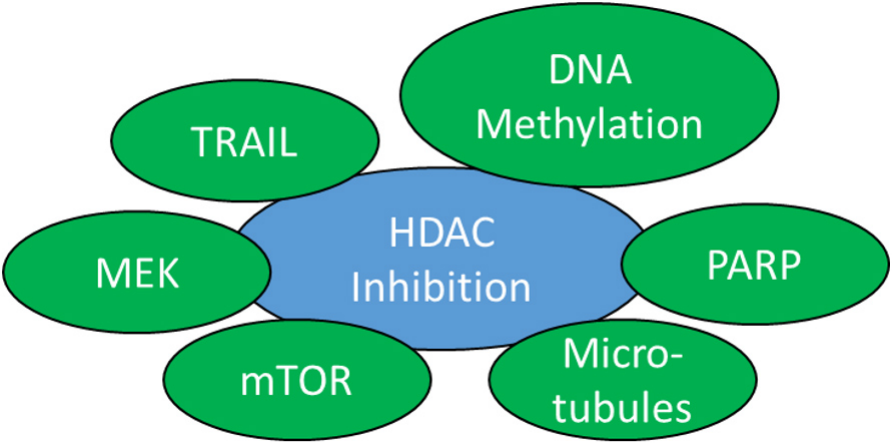
Suitable targets for combination therapies with HDAC inhibitors in CRPC. HDAC: Histone deacetylase; CRPC: castration-resistant prostate cancer; MEK: MAPK/ERK kinase; TRAIL: TNF-related apoptosis-inducing ligand.

The detailed knowledge of the mechanisms of action of HDAC inhibitors in prostate cancers can be applied for combination therapies together with other suitable drugs against prostate cancer to achieve improved anticancer responses [Table 3, Figures 1 and 3]. DNA-based mechanisms appear to be promising drug targets for combination partners of HDAC inhibitors. Inhibition of DNA methylation was positively correlated with HDAC inhibition and sodium butyrate in combination with the DNA methyltransferase inhibitor 5-aza-2’-deoxycytidine significantly enhanced histone H4 acetylation and induced AR gene re-expression in androgen-independent DU145 prostate cancer cells. Moreover, it led to G2/M cell cycle arrest and reduced toxicity to non-malignant cells^[48]^. More recently, panobinostat was investigated in combination with the DNA methylation inhibitor hydralazine in various prostate cancer cells. The combination therapy was particularly active against androgen-independent DU145 prostate cancer cells, induced apoptosis, and reduced colony formation, invasion, and migration^[49]^. Hence, the combination of panobinostat with hydralazine has the potential to block prostate cancer progression and might be a suitable therapy in future clinical studies.

**Table 3:** HDAC inhibitors in combination with other drugs against CRPC

**HDAC inhibitor**	**Combination partner (function)**	**Outcome**
Sodium butyrate^[48]^	5-Aza-2’-deoxycytidine (DNA methyltransferase inhibitor)	Enhanced histone H4 acetylation, induced AR gene re-expression, G2/M cell cycle arrest, reduced non-malignant cell toxicity
Panobinostat^[49]^	Hydralazine (DNA methylation inhibitor)	induced apoptosis, reduced colony formation, invasion, and migration
Vorinostat^[52]^	Olaparib (PARP inhibitor)	Synergistic reduction of cell viability, induced apoptosis, suppressed DNA repair, BRCA1 and RAD51 expression downregulated
Vorinostat^[53]^	Veliparib (PARP inhibitor)	Synergistic effects on cell viability (BRCA1-mutant DU145), apoptosis induction, DNA damage production, BRCA1 suppression/degradation, suppressed UHRF1
Vorinostat^[54]^	Docetaxel (microtubules stabilizer)	Increased tubulin acetylation and bundling, suppressed AR and Bcl-2 expression, suppressed nuclear AR translocation and AR signaling
Trichostatin A^[55]^	Bicalutamide (anti-androgen) and finasteride (5α-reductase inhibitor)	Synergistic apoptosis induction
Panobinostat^[57]^	Dovitinib (multi-RTK inhibitor)	No improved effects
Ricolinostat^[58]^	Selumetinib (MEK inhibitor) and paclitaxel (microtubules stabilizer)	Synergistic growth inhibition and apoptosis induction, suppression of KLK2 and DUSP1, increased AR accumulation in cytoplasm
Valproic acid^[62-64]^	Everolimus (mTOR inhibitor) and IFNα	Increased cell growth inhibition, suppression of EGFR (epidermal growth factor receptor), ERK1, and ERK2, increased acetyl-H3
Panobinostat^[67]^	Everolimus (mTOR inhibitor)	Myc-CaP cell growth inhibition, suppressed clonogenic survival and G0/G1 cell cycle arrest, increased p21 and p27 expression; *in vivo* suppression of tumor growth, AR, HIF-1α, miR-20a, miR-21
Romidepsin, Entinostat^[70]^	Adenoviral TRAIL gene therapy	Restored CAR surface expression, augmented TRAIL-mediated caspase activity

PARP: Poly-ADP-ribosyl-polymerase; RTK: receptor tyrosine kinase; MEK: MAPK/ERK kinase; IFNα: interferon-α; mTOR: mammalian target of rapamycin; EGFR: epidermal growth factor receptor; ERK: extracellular signal-regulated kinase; HIF: hypoxia inducible factor; miR: microRNA; CAR: coxsackie adenovirus receptor; TRAIL: TNF-related apoptosis-inducing ligand.

Poly-ADP-ribosylpolymerase (PARP) is an enzyme involved in DNA repair. And the PARP inhibitor olaparib performed well in advanced clinical trials with CRPC patients who have a BRCA mutation, which led to the approval of olaparib for the treatment of BRCA-mutant prostate cancer^[50,51]^. The combination of vorinostat with olaparib synergistically reduced prostate cancer cell viability, induced apoptosis in DU145 and PC3 prostate carcinoma cells, and suppressed DNA repair as well as DNA repair protein expression (BRCA1 and RAD51) in DU145 cells^[52]^. The combination of vorinostat with the PARP inhibitor veliparib also revealed synergistic effects on prostate cancer cell viability (in particular, on the viability of BRCA1-mutant DU145 cells), apoptosis induction, DNA damage production, and BRCA1 suppression. The vorinostat/veliparib combination therapy suppressed BRCA1 by downregulation of the UHRF1 protein, which forms a stable DNA repair protein complex with BRCA1, and suppression of UHRF1 led to degradation of BRCA1^[53]^. Thus, the combination of vorinostat with PARP inhibitors such as olaparib and veliparib appears to be a promising combination therapy for future clinical trials with CRPC patients.

Vorinostat was also studied in combination with the microtubule-stabilizing anticancer taxane drug docetaxel, and the combination showed synergistic growth inhibitory effects on 22Rv1 and VCaP CRPC cells. This combination therapy suppressed AR and Bcl-2 expression, AR translocation into the prostate cancer cell nucleus, and AR signaling. The reduced accumulation of the AR in the nucleus was based on increased acetylation and bundling of tubulin in cells treated with the HDAC inhibitor vorinostat and the microtubule stabilizer docetaxel^[54]^. Another study targeting AR signaling comprised the combination treatment of androgen-accustomed DuCaP-N prostate cancer cells with the HDAC inhibitor TSA, the anti-androgen bicalutamide, and the 5α-reductase inhibitor finasteride. The combination of TSA with bicalutamide and finasteride led to synergistic apoptosis induction in the DuCaP-N cells^[55]^.

Protein kinases are valuable targets for new anticancer drugs. Enhanced receptor tyrosine kinase (RTK) signaling was observed in advanced prostate cancers and appeared as a suitable drug target for targeted therapies^[56]^. Thus, the combination of HDAC inhibitors with TK inhibitors seems to be reasonable. However, the combination of the multi-RTK inhibitor dovitinib (TKI258) with panobinostat did not show improved anticancer effects on prostate cancer cells since dovitinib appeared to be particularly inactive in the tested prostate cancer cells^[57]^. MAPK/ERK kinase (MEK, a serine/threonine/tyrosine kinase) inhibitors suppress the Ras-MAPK signaling pathway in cancers, and a series of anticancer active MEK inhibitors was developed^[58]^. The MEK inhibitor selumetinib was studied in combination with the HDAC6 inhibitor ricolinostat in various CRPC cell lines (PC3, DU145, and 22Rv1). While the combination treatment revealed synergistic effects in all three cell lines, 22Rv1 cells were particularly sensitive in terms of growth inhibition. A suppression of the AR target genes KLK2 and DUSP1 was observed in treated 22Rv1 cells as well as increased AR accumulation in the cytoplasm. Addition of the taxane paclitaxel to the ricolinostat/selumetinib combination led to synergistic growth inhibition and increased apoptosis induction in all three prostate cancer cell lines, which coincides well with the aforementioned anti-prostate cancer effects of the combined vorinostat/docetaxel treatment^[59]^.

Akt and its downstream factor mTOR (mammalian target of rapamycin) are well established anticancer targets of the phosphatidylinositol 3-kinase (PI3K) signaling pathway^[60]^. Various mTOR inhibitors such as rapamycin, everolimus, and temsirolimus were developed and approved by the FDA^[61]^. The anticancer activities comprising inhibition of prostate cancer cell growth, migration, and invasion of HDAC inhibitors such as valproic acid were increased when combined with everolimus^[62,63]^. Furthermore, the activity of valproic acid plus everolimus was augmented by addition of low-dose interferon-α (IFNα), and this triple-drug therapy was superior to single-drug therapy in terms of prostate tumor cell growth inhibition and dissemination. Intracellular signaling of PC3 cells was downregulated based on suppression of EGFR (epidermal growth factor receptor), ERK1, and ERK2, while acetyl-H3 levels were increased in treated cells^[64]^. Panobinostat was also studied in combination with everolimus in prostate cancer. For this reason, the *c*-Myc expressing Myc-CaP prostate cancer cell line was applied because c-Myc expression reduced the activity of the mTOR inhibitor rapamycin in prostate cancer^[65,66]^. The combination of panobinostat with everolimus exhibited significant Myc-CaP cell growth inhibition, while the combined treatment of Myc-CaP cells at non-cytotoxic doses of 10 nM for each compound suppressed clonogenic survival and induced G0/G1 cell cycle arrest associated with increased expression of the cyclin-dependent kinase inhibitors p21 and p27. In addition, combined panobinostat (10 mg/kg, i.p.) and everolimus (10 mg/kg, p.o.) treatment for 15 days (QD × 7 schedule) showed reduction of tumor proliferation and tumor volume in mice bearing androgen-sensitive as well as castration-resistant Myc-CaP tumors. The combination treatment blocked AR and HIF-1α transcriptional activities both *in vitro* and *in vivo* and downregulated the oncogenic microRNAs (oncomirs) miR-20a and miR-21 *in vivo*, which were associated with AR/hypoxia and *c*-Myc/hypoxia signaling pathways^[67]^.

Virus therapies have become interesting tools for gene therapies of tumor diseases and adenoviral TRAIL gene therapy caused signs of apoptosis in the examined prostates of prostate cancer patients^[68]^. Since gene suppression in cancer cells can be reversed by HDAC inhibitors, the combination of gene therapy with HDAC inhibitors seems to be feasible. The HDAC inhibitors romidepsin and MS-275 increased the effects of adenoviral TRAIL gene therapy on castration-sensitive LNCaP prostate cancer cells without toxicity to non-malignant prostate epithelial cells^[69]^. Based on this finding, the combination of TRAIL gene therapy with romidepsin or MS-275 in C4-2B CRPC cells was studied. Decreased coxsackie and adenovirus receptor (CAR) expression was correlated with increased tumorigenicity and metastasis formation in the LNCaP-derived human prostate cancer subline C4-2B, however, both aforementioned HDAC inhibitors restored CAR surface expression and augmented TRAIL-mediated caspase activity based on enhanced adenoviral transduction efficacy when combined with TRAIL gene therapy^[70]^.

### New HDAC inhibitors

**Figure 4:**
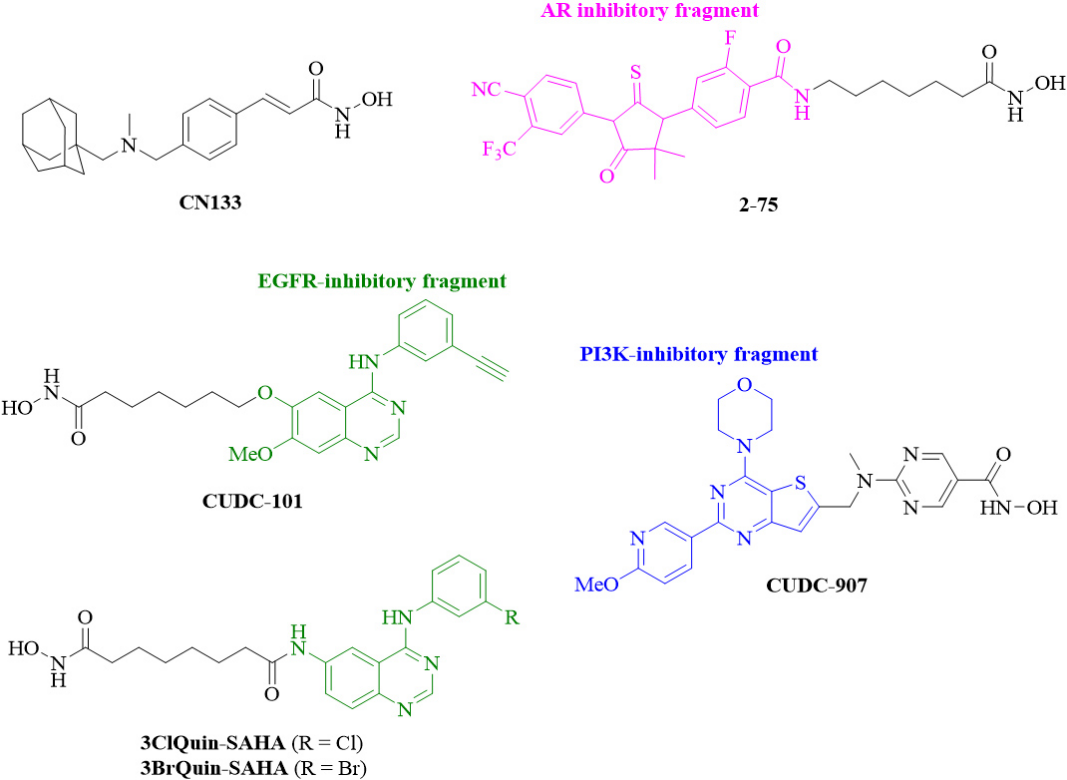
Structures of new HDAC inhibitors with promising activities against CRPC. HDAC: Histone deacetylase; CRPC: castration-resistant prostate cancer; AR: androgen receptor; EGFR: epidermal growth factor receptor; PI3K: phosphatidylinositol 3-kinase.

Aside from combination of approved HDAC inhibitors with other anticancer drugs, the development of new HDAC inhibitors with improved anticancer properties turned out to be a reasonable strategy to tackle CRPC [Table 4, Figure 4].

**Table 4:** New HDAC inhibitors with promising anti-CRPC activities

**Compd.**	**Structural motif(s)**	***In vitro*** ** activity**	***In vivo*** ** activity**
CN133^[71]^	Hydroxamic acid, adamantyl cap	Inhibition of HDAC1-3; 100 times more active than SAHA (22Rv1 cells), inhibition of cell migration, invasion and AR signaling	Tumor growth and weight reduction by 50% (22Rv1)
2-75^[74,75]^	Hydroxamic acid, AR-targeting enzalutamide-type cap	HDAC inhibitory activity, induced p21, higher acetyl-tubulin levels (based on stronger HDAC6 inhibition) than SAHA, suppressed Hsp90 and AR/AR-V7	Improved long-term tumor growth inhibition, enhanced apoptosis, reduced nuclear AR accumulation (LNCaP)
CUDC-101^[77]^	Hydroxamic acid, EGFR/HER2-targeting erlotinib-type anilinoquinazoline cap	Suppressed AR, AR-V7, and HER2	Significant tumor growth inhibition without weight loss (22Rv1)
3ClQuin-SAHA, 3BrQuin-SAHA^[84]^	Hydroxamic acid, EGFR-targeting gefitinib-type anilinoquinazoline cap	Higher antiproliferative activity than gefitinib (DU145 cells), HDAC inhibition, EGFR inhibition, mTOR suppression	-
CUDC-907 (fimepino-stat)^[86]^	Hydroxamic acid, PI3K-targeting thienopyrimidine cap	High growth inhibitory activity, inhibition of HDACs and PI3K, apoptosis induction, increased Bim, suppressed Mcl-1 and Bcl-xL, suppressed DNA repair and DNA damage response proteins (Wee1, CHK1, RRM1, and RRM2), suppressed *c*-Myc	Tumor growth inhibition by 60% without weight loss (LuCaP 35CR patient-derived mouse xenografts)

AR: Androgen receptor; EGFR: epidermal growth factor receptor; HER2: human epidermal growth factor receptor 2; mTOR: mammalian target of rapamycin; PI3K: phosphatidylinositol 3-kinase.

The adamantyl-capped HDAC inhibitor CN133 was superior to vorinostat in terms of Class I HDAC1, -2, and -3 inhibition (IC_50_ = 0.6, 2, and 0.3 nM for CN133; 4, 11, and 3 nM for vorinostat), while vorinostat was more active against HDAC6 (IC_50_ = 2 nM) than CN133 (IC_50_ = 4.1 nM). CN133 was 100 times higher antiproliferative (IC_50_ = 10 nM) in 22Rv1 CRPC cells than vorinostat (IC_50_ = 1 μM). CN133 inhibited CRPC cell migration and invasion and suppressed AR signaling. Mice bearing 22Rv1 CRPC were treated with CN133 (1 mg/kg), which reduced tumor volume and tumor weight by 50% in comparison with placebo group mice^[71]^.

Hybrid molecules targeting two or more anticancer targets can possess higher anticancer activities accompanied by reduced drug-drug interactions and less complex pharmacokinetics^[72,73]^. Several chimeric HDAC inhibitors with dual or multimodal activities were reported over the last years^[29,30]^. Compound 2-75 is a promising enzalutamide hybrid with HDAC inhibitory activity, which induced p21, led to higher acetyl-tubulin levels (based on stronger HDAC6 inhibition) than vorinostat, and suppressed Hsp90 and AR protein levels in C4-2 prostate cancer cells^[74]^. Based on these results, deeper studies of 2-75 in CRPC were carried out. Compound 2-75 suppressed DHT-induced AR transcriptional activity and AR translocation to the nucleus stronger than enzalutamide. In addition to AR, the mutant AR-V7 was also downregulated by 2-75 in prostate cancer cells in a proteasome-dependent way, indicating enhanced AR degradation in 2-75-treated cells. *In vivo* experiments with LNCaP tumor models revealed that 2-75 treatment (10 mg/kg, intratumoral injection twice weekly) had tumor growth inhibitory activity similar to enzalutamide, but, in the long run (after Day 24), 2-75 displayed improved tumor growth inhibition when compared with enzalutamide. The *in vivo* activity of 2-75 was accompanied by increased apoptosis induction and suppressed AR nuclear accumulation in the tumor bodies of treated mice^[75]^.

The aforementioned receptor tyrosine kinases such as EGFR are excellent targets for the design of new HDAC/EGFR inhibitors. Compound CUDC-101 combines an HDAC inhibitory fragment with an EGFR inhibitory scaffold derived from the approved anticancer active EGFR inhibitor erlotinib and inhibited HDAC, EGFR, and HER2^[76]^. In CRPC cells, CUDC-101 suppressed full-length AR as well as the variant AR form AR-V7, upregulated p21, and downregulated HER2/NEU. In castrated mice bearing aggressive 22Rv1 CRPC tumors, CUDC-101 (50 μg/kg/day for 14 days) inhibited tumor growth significantly without measurable weight loss of treated mice^[77]^.

Erlotinib and CUDC-101 have certain drawbacks, which warranted further research efforts. The ethinylphenyl residue of erlotinib can be activated by cytochrome P450 enzymes leading to oxidized phenol and quinone compounds with certain toxicity potential^[78]^. Indeed, erlotinib was reported to increase the risk of lethal gastrointestinal tract perforations in cancer patients taking corticosteroids or ciprofloxacin^[79]^. A clinical phase 1 study of CUDC-101 together with chemoradiation in head and neck squamous cell carcinoma (HNSCC) patients reported that five out of twelve HNSCC patients (i.e., 41%) had to stop the therapy due to adverse events^[80]^. In addition, CUDC-101 was shown to be a substrate of the cell-detoxifying ABC transporters ABCB1 (P-gp) and ABCG2 (BCRP) leading to resistance to CUDC-101 treatment, whereupon the combination with P-gp and/or BCRP inhibitors was suggested in order to avoid CUDC-101 resistance formation^[81]^. In contrast to that, the approved EGFR inhibitor gefitinib reversed P-gp- and BCRP-mediated drug resistance *in vitro* and *in vivo*^[82,83]^. Hence, gefitinib is a promising conjugation partner, and chimeric HDAC inhibitors with gefitinib-derived cap scaffolds based on halogen-substituted aniline rings were prepared. The chimeric compounds 3ClQuin-SAHA and 3BrQuin-SAHA showed 3-4 times higher growth inhibitory activity (IC_50_ = 3.23 μM for 3ClQuin-SAHA and 3.53 μM for 3BrQuin-SAHA) against DU145 CRPC cells than gefitinib (IC_50_ = 11.9 μM); however, vorinostat (IC_50_ = 0.68 μM) was still more antiproliferative in these prostate cancer cells. Nevertheless, 3ClQuin-SAHA and 3BrQuin-SAHA combined EGFR inhibitory activity with HDAC inhibition, suppressed EGFR expression comparable to vorinostat, showed only marginal unspecific toxicities, induced apoptosis in DU145 cells, and inhibited angiogenesis^[84]^. Hence, these chimeric compounds can be suitable anticancer drug candidates in prostate cancer therapy in terms of reduced erlotinib (and vorinostat) toxicity and resistance formation.

CUDC-907 (fimepinostat) is another promising HDAC/kinase inhibitor, which was designed to target HDAC enzymes and the kinase PI3K^[85]^. Based on previous reports describing the synergistic effects of CUDC-907 in various cancer models and since PI3K signaling also plays an eminent role for CRPC development (see the aforementioned role of mTOR inhibitors), CUDC-907 was investigated in prostate cancer models. CUDC-907 showed excellent growth inhibitory activities in a panel of eight prostate cancer cell lines with IC_50_ values between 2 and 17.4 nM, inhibited HDACs and PI3K signaling, and induced apoptosis in a dose-dependent way associated with increased pro-apoptotic Bim and suppressed anti-apoptotic Mcl-1 and Bcl-xL expression in 22Rv1 CRPC cells. In addition, CUDC-907 treatment led to enhanced DNA damage due to downregulated DNA damage response proteins (Wee1, CHK1, RRM1, and RRM2). The protein expression of the oncoprotein *c*-Myc, which regulates the mentioned apoptosis factors and DNA damage response proteins, was also suppressed by CUDC-907 and, thus, *c*-Myc suppression plays a key role in the anti-prostate cancer mode of action of CUDC-907. Finally, CUDC-907 (100 mg/kg/day, p.o.) was tested in castration-resistant LuCaP 35CR mouse xenografts and inhibited *in vivo* tumor growth by ca. 60% while no weight loss was detected^[86]^. More recently, a phase 2 study of oral CUDC-907 for the treatment of relapsed/refractory diffuse large and high-grade B-cell lymphoma (DLBCL and HGBL) patients with enhanced Myc-expression and/or altered *MYC* gene constitutions (e.g., altered gene composition by translocation) was published revealing an overall response rate of 22% in the MYC-altered disease patients^[87]^. Hence, patients enrolled for future prostate cancer clinical studies with oral CUDC-907 should be tested for their MYC-status. In contrast to CUDC-101, CUDC-907 was not a substrate of P-gp (ABCB1) transporters, and its antiproliferative activity was conserved in P-gp-expressing tumor cells. However, CUDC-907 was still efficiently inactivated by BRCP (ABCG2) transporters, similar to CUDC-101, and combinations with BCRP inhibitors were recommended to keep the high anticancer activity of CUDC-907^[88]^.

## CONCLUSION

HDAC inhibitors exhibit reasonable *in vitro* and *in vivo* activities against CRPC. Clinical trials have shown that monotherapy with HDAC inhibitors cannot be recommended now for phase 3 studies due to low efficacy and considerable toxicity in CRPC patients. However, the combination of HDAC inhibitors with certain anticancer drugs such as taxanes and anti-androgens appeared promising based on the data from clinical studies. In preclinical studies, DNA methylation inhibitors, PARP inhibitors, various protein kinase and mTOR inhibitors, interferon, and TRAIL gene therapy appeared to be suitable combination partners for HDAC inhibitors, which should be considered for future clinical studies with CRPC patients.

Various new HDAC inhibitors have emerged over the last years, which displayed significant activity against CRPC in preclinical studies. Many of these new HDAC inhibitors were designed to target non-HDAC proteins such as protein kinases or the AR in addition to HDAC enzymes. Among them, CUDC-101 and CUDC-907, which are HDAC/kinase inhibitors, are striking examples that have already entered clinical trials. The activities of these two drug candidates were thoroughly studied in preclinical CRPC models giving hints at strengths and weaknesses of multi-targeting HDAC inhibitors. Strong CRPC growth inhibition accompanied by good drug tolerance in mice were contrasted by toxicity in patients and resistance formation based on the expression of ABC transporters in tumor cells. Efforts to eliminate the drawbacks of CUDC-101 and CUDC-907 are already in progress and the first preclinical results of new HDAC/kinase inhibitory derivatives are available. In addition, subgroups of CRPC patients with increased Myc levels should be considered for future clinical trials with CUDC-907 since lymphoma patients with elevated Myc responded well to this sophisticated HDAC inhibitor.

The drug developments described in this work are based on the elucidation of the mechanisms of action of first-generation HDAC inhibitors, the design of new therapy regimens, and the synthesis of new HDAC inhibitors with improved anticancer properties. The close interplay of clinicians, tumor biologists, and synthetic chemists has led to promising outcomes in the field of CRPC research over the last years, and interdisciplinary work will continue to help HDAC inhibitor-based therapy to play a prominent role as efficient and well-tolerated CRPC treatment in the future.

## DECLARATIONS

### Copyright

© The Author(s) 2022.
